# COVID-19's impact on lung tissue: A case report

**DOI:** 10.1016/j.ijscr.2022.106905

**Published:** 2022-03-01

**Authors:** Connor Crudeli, Brian Zilberman, Jennifer Williams, Jennifer Burg, David Shersher

**Affiliations:** a Cooper Medical School of Rowan University, 401 Broadway, Camden, NJ 08103, USA; b Cooper University Hospital, Department of Surgery, 1 Cooper Plaza, Camden, NJ 08103, USA; c Cooper University Hospital, Department of Surgery, Thoracic Surgery, 1 Cooper Plaza, Camden, NJ 08103, USA

**Keywords:** COVID-19, Coronavirus Disease 2019, ICU, Intensive Care Unit, COVID-19, Empyema, Pneumonia, Pulmonary microthrombi, Case report, Thoracic surgery

## Abstract

**Introduction:**

The novel coronavirus has spread globally, however, there continues to be little information regarding management, treatment, and complications encountered by infected patients. Prior to COVID-19, guidelines had been well established for managing empyema, however, evidence is lacking for such patients possessing a COVID-19 infection. In the spirit of collaborative knowledge, we endeavor to present a COVID-19 case from our tertiary care institution.

**Case presentation:**

A 59-year-old Caucasian male with a past medical history of chronic obstructive pulmonary disease and hypertension was transferred to our hospital for escalation of care of COVID pneumonia. Pharmaceutical treatment included an IL-6 inhibitor (tocilizumab). The patient's hospital course was complicated by superimposed bacterial pneumonia with development of a loculated pleural empyema. On day 57, a left anterolateral muscle-sparing thoracotomy and complete pulmonary decortication was performed. The patient made a successful recovery.

**Clinical discussion:**

This patient's vascular dysfunction associated with shunting secondary to pulmonary microthrombi, provides rationale for the liberal use of therapeutic anticoagulation in COVID patients. The superimposed bacterial pneumonia raises concerns over the use of tocilizumab in COVID-19 patients. It is necessary to understand whether current guidelines will need to be amended for the treatment of coagulopathies to avoid pulmonary vascular dysfunction.

**Conclusion:**

Thoracic surgery can be carried out safely, both for patients and practitioners, during the pandemic. Microvascular occlusions within the pulmonary vasculature contribute to the severe hypoxia and need for anticoagulation in severe COVID-19 cases. Clinical pathways for common clinical presentations, such as empyema, may need to be re-evaluated during this global crisis.

## Highlights

• Varying pathology observed in COVID-19 patients undergoing pulmonary resection
• Vascular dysfunction associated with shunting secondary to pulmonary microthrombi
• Microvascular occlusions contribute to severe hypoxia and need for anticoagulation.
• Thoracic surgery can be carried out safely during the pandemic.
• Clinical pathways for COVID-19 patients may need to be re-evaluated.

## 1 Introduction

The novel coronavirus (COVID-19) has spread globally to every continent since its first appearance late last year in the City of Wuhan, Hubei province of China. Despite the global incidence of disease totaling over 29,000,000 cases, 936,156 deaths and over 260 days since the first reported case, there continues to be limited information available to the scientific and medical community regarding management, treatment, histopathology, and complications encountered when caring for patients infected with this disease [1]. This poor understanding of the pathophysiological changes includes the intrathoracic space, where COVID-19 may manifest as pneumonia, lung abscess, or empyema. Guidelines are well established for managing non-COVID-19-infected patients with empyema, but there is little data to guide clinical efforts in a similar cohort of patients infected by COVID-19 [2]. Herein we present a complex clinical course following COVID-19 infection with associated intrathoracic illness.

This case report has been reported in line with the SCARE 2020 criteria [3].

## 2 Case presentation

A 59-year-old male with a past medical history of chronic obstructive pulmonary disease, hypertension, gastroesophageal reflux disease, intracranial bleed, posterior pseudo-meningocele as well as schizophrenia was transferred to our tertiary care hospital for escalation of care on 3/30/2020 with a known COVID-19 infection based on PCR test. The patient experienced rapid worsening respiratory failure with acute respiratory distress syndrome, septic shock related to bacterial pneumonia, cytokine storm syndrome requiring intubation and initiation of multiple vasopressors. Shortly after arrival he was placed in a prone position as part of a lung protective protocol. His pharmaceutical treatment involved multiple therapies including azithromycin, vancomycin, cefepime, hydroxychloroquine, lopinavir/ritonavir, and the interleukin 6 inhibitor tocilizumab.

**Fig. 1:**
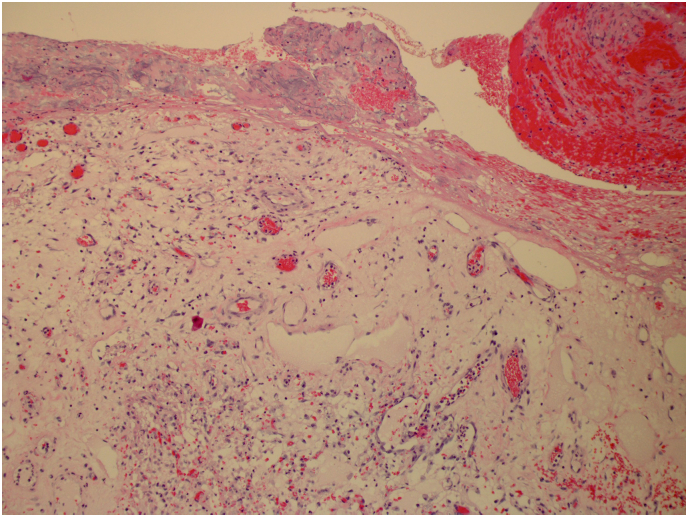
Inflamed fibrinous exudate of pleural surface consistent with empyema.

**Fig. 2:**
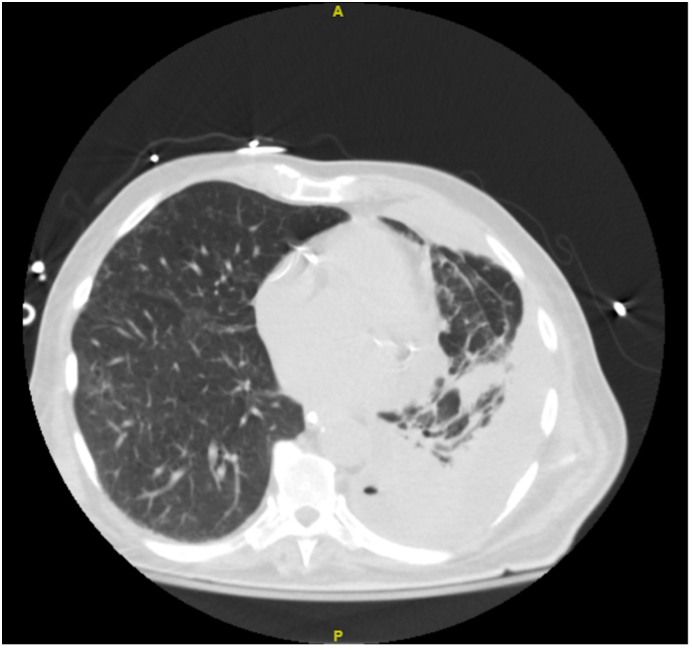
Computed tomography chest showing empyema in the setting of dense left lower lobe air space disease.

After clinical stabilization, he was later transferred to the general inpatient floor on hospital day 16 and subsequently tested negative via COVID-19 by PCR. However, in the following days, he once again tested positive for COVID-19 after he redeveloped respiratory distress requiring transfer back to the intensive care unit (ICU). Following a failed attempt at extubating, the patient underwent a tracheostomy and percutaneous endoscopic gastrostomy tube. The patient's hospital course was further complicated by superimposed bacterial pneumonia and associated left empyema infected by *Pseudomonas aeringosa* (Fig. 1, Fig. 2). After placement of a chest tube, the patient developed a complex hemothorax thought to be a result of a combination of tissue plasminogen activation instillations and systemic therapeutic anticoagulation for deep vein thrombosis.

**Fig. 3:**
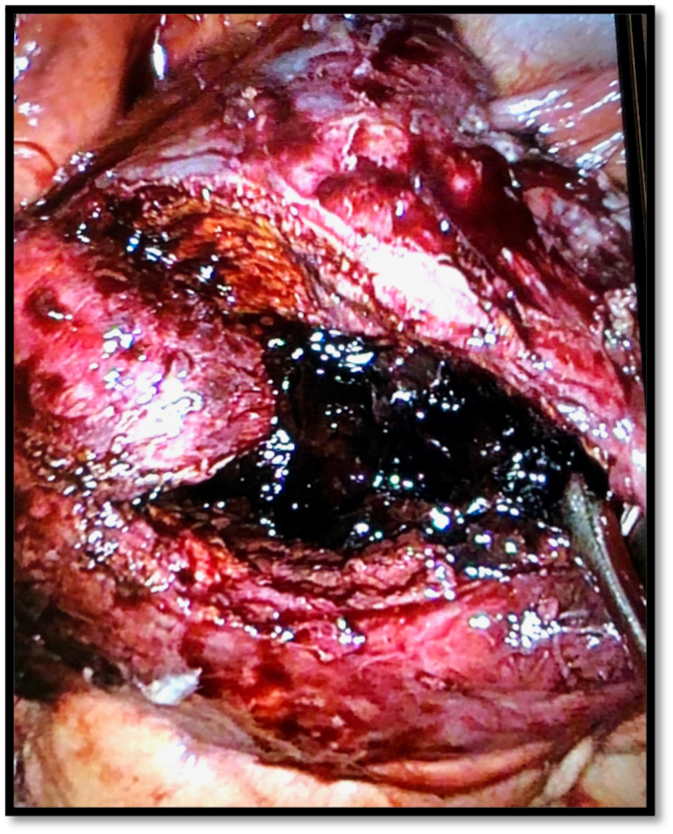
Intra-operative gross image of necrotic left lower lobe, basilar segment.

After an extensive period of nonoperative management, the patient was evaluated by the thoracic surgery team due to failure to improve clinically. On hospital day 57, following a multidisciplinary discussion, the patient underwent a left anterolateral muscle-sparing thoracotomy and complete pulmonary decortication. Due to the extensive intrapleural adhesions and associated necrotic left lower lobe of the lung that was fused to the diaphragm (Fig. 3), the patient underwent multiple left lower lobe therapeutic extended wedge en bloc with diaphragm and primary diaphragmatic repair. The operation was challenging with respect to impressively thick adhesions as well as 250 mL of associated hemothorax and multiple purulent lung abscesses. During the operation, areas of non-viable, necrotic lung tissue were completely resected. The specimens obtained were sent to pathology for further analysis. The patient recovered well after surgery and eventually was transferred to an outside facility for rehabilitation.

## 3 Discussion

It is still unclear how COVID-19 infection interplays with intrathoracic pathology and co-infection affected both the pleural space as well as the lung parenchyma. The aforementioned case illustrates well the complex multidisciplinary effort necessary to care for a COVID-19 patient with multiorgan system failure.

**Fig. 4:**
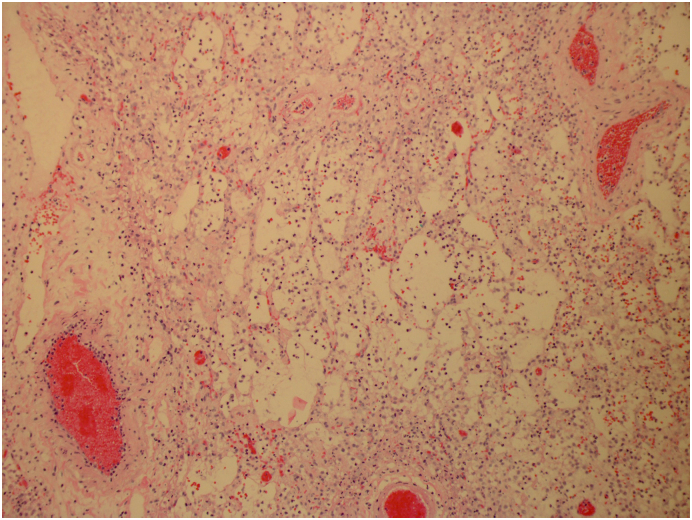
Microscopy demonstrating infarcted pulmonary parenchyma and ghosted outlines of alveolar septa.

**Fig. 5:**
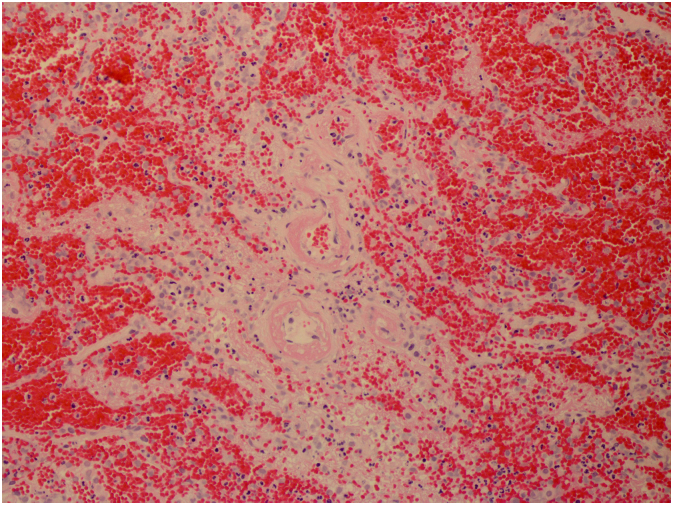
Mural fibrinoid necrosis of small pulmonary vessels consistent with vasculitis.

**Fig. 6:**
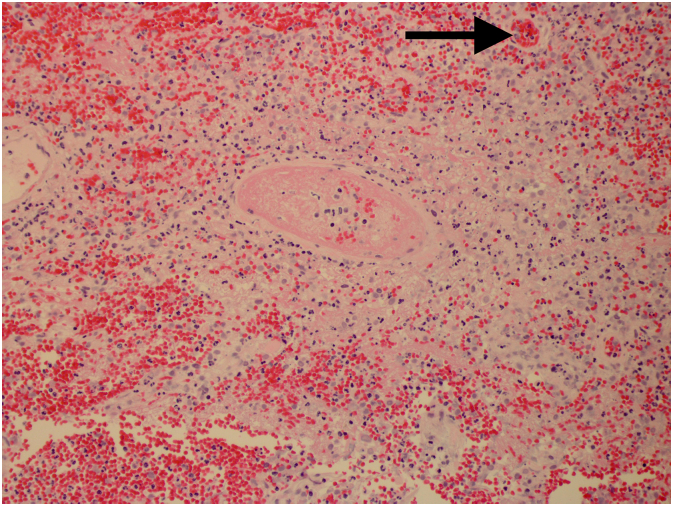
A fibrin thrombus within a small pulmonary blood vessel.

At our institution, like many others, we see significant impact of the overall illness in the chest with varying degrees of pathology [4]. Decortications are still performed for bacterial empyema, however when coupled with previous or ongoing COVID-19 infection, are subjectively more likely to require parenchymal resection for necrotic lung segments associated with lung abscess and microthrombi. This is consistent with other reports in literature as recent published cases have reported common histopathological findings of lung specimens in patients with a COVID-19 positive diagnosis [4]. These findings describe the presence of infiltrating plasma cells and macrophages with septal thickening/disruption and additional fibrinous proliferation [5], [6], [7]. Interestingly, our patient's pathology demonstrated a less commonly reported finding of vascular dysfunction associated with shunting secondary to pulmonary microthrombi. Histopathological evidence demonstrates other unique findings associated with COVID-19 including infarcted pulmonary parenchyma demonstrating ghosted outlines of alveolar septa (Fig. 4), small vessel vasculitis with mural fibrinoid necrosis (Fig. 5), and a fibrin thrombus in a small pulmonary vessel (Fig. 6). If indeed microthrombi impact ischemia to a lung segment and contribute to lung abscess formation, perhaps this provides pulmonary rationale for the liberal use of therapeutic anticoagulation in COVID-19 patients [8]. These findings reinforce the need for pharmacological thrombosis prophylaxis in all COVID-19 patients admitted to the ICU and are consistent with the findings of a recent study that found a 31% incidence of thrombotic complications in this patient population [9].

Literature regarding COVID-19 infected patients is limited and even less available concerning longstanding COVID infections such as described in this case presentation. Prolonged and complicated ICU stays involving the use of extensive pharmacological treatment and surgical interventions may introduce additional risks during recovery.

The superimposed bacterial pneumonia experienced during this patient's stay raises concerns over the use of immunosuppressive drugs such as tocilizumab in COVID-19 patients as well as optimal timing of operative interventions. The use of tocilizumab in COVID-19 patients complicates treatment as the cytokine activation syndrome associated with its use may suppresses common signs of sepsis [10]. Previous studies involving tocilizumab in rheumatological diseases have demonstrated an increased risk for serious bacterial infections [11], [12], [13], [14]. Further investigation is needed in order to address the benefits and possible risk factors associated tocilizumab administration and superimposed bacterial pneumonia in COVID-19 patients.

Additionally, as the population of previously or currently infected COVID-19 patients undergoing thoracic procedures increases, it is necessary to understand whether current guidelines will need to be adjusted to include treatment of coagulopathies in order to avoid pulmonary vascular dysfunction. Deciding appropriate management for patients with COVID-19 poses a major challenge to the global healthcare community, and a better understanding of pulmonary sequelae of this disease is paramount. The novel virus necessitates a comprehensive review of current standards and guidelines in order to achieve more successful outcomes and minimize complications and lengthy hospital stays.

## 4 Conclusions

Thoracic surgery can be carried out safely, both for patients and practitioners, during the pandemic. Microvascular occlusions within the pulmonary vasculature contribute to the severe hypoxia and need for anticoagulation in severe COVID-19 cases. Clinical pathways for common clinical presentations, such as empyema, may need to be re-evaluated during this global crisis.

## Sources of funding

The authors declare no funding source for this research.

## Ethical approval

This case report is exempted by Cooper University Hospital Institutional Review Board.

## Consent

Written informed consent was obtained from the patient for publication of this case report and accompanying images. A copy of the written consent is available for review by the Editor-in-Chief of this journal on request.

## Author contribution

David Shersher MD and Brian Zilberman MD developed the study concept. Connor Crudeli BA conducted the literature research and prepared the initial manuscript. Connor Crudeli BA, Brian Zilberman MD, Jennifer Williams MD, Jennifer Burg MD, and David Shersher MD revised the manuscript. Connor Crudeli BA organized, prepared, and submitted the final version of the manuscript. David Shersher MD performed the surgery and is responsible for providing intraoperative imaging. The histological analysis was performed by William Rafferty MD, Cooper Univeristy Hospital Department of Pathology.

## Research registration

N/A.

## Guarantor

Connor Crudeli BA.

## Provenance and peer review

Not commissioned, externally peer-reviewed.

## Declaration of competing interest

The authors have no financial, consultative, institutional, and other relationships that might lead to bias or conflict of interest.
